# The Relationship between Telomere Length and Gestational Weight Gain: Findings from the Mamma & Bambino Cohort

**DOI:** 10.3390/biomedicines10010067

**Published:** 2021-12-30

**Authors:** Andrea Maugeri, Roberta Magnano San Lio, Maria Clara La Rosa, Giuliana Giunta, Marco Panella, Antonio Cianci, Maria Anna Teresa Caruso, Antonella Agodi, Martina Barchitta

**Affiliations:** 1 Department of Medical and Surgical Sciences and Advanced Technologies “GF Ingrassia”, University of Catania, Via S.Sofia, 87, 95123 Catania, Italy; andrea.maugeri@unict.it (A.M.); roberta.magnanosanlio@phd.unict.it (R.M.S.L.); mariaclara.larosa@unict.it (M.C.L.R.); martina.barchitta@unict.it (M.B.); 2 Obstetrics and Gynecology Unit, Department of General Surgery and Medical Surgical Specialties, University of Catania, Via S. Sofia, 78, 95123 Catania, Italy; giunta.giuliana@studium.unict.it (G.G.); mpanella@unict.it (M.P.); acianci@unict.it (A.C.); 3 Cytogenetic Laboratory, Azienda Ospedaliero Universitaria Policlinico “G. Rodolico—San Marco”, Via S. Sofia, 78, 95123 Catania, Italy; m.caruso@ao-ve.it

**Keywords:** pregnancy, aging, telomere, weight gain, body mass index

## Abstract

Inadequate gestational weight gain (GWG) affects a growing number of pregnancies, influencing intrauterine environment and long-term health. Uncovering molecular mechanisms associated with GWG could be helpful to develop public health strategies for tackling this issue. Here, our study aimed to understand the relationship of DNA telomere length with weigh gain during pregnancy, using data and samples from the ongoing prospective “Mamma & Bambino” study (Catania, Italy). GWG was calculated according to the Institute of Medicine (IOM) guidelines. Relative telomere length was assessed by real-time quantitative polymerase chain reaction in 252 samples of maternal leucocyte DNA (mlDNA) and 150 samples of cell-free DNA (cfDNA) from amniotic fluid. We observed that relative telomere length of mlDNA seemed to weakly increase with GWG. In contrast, telomere length of cfDNA exhibited a U-shaped relationship with GWG. Women with adequate GWG showed longer telomere length than those who gained weight inadequately. Accordingly, the logistic regression model confirmed the association between telomere length of cfDNA and adequate GWG, after adjusting for potential confounders. Our findings suggest an early effect of GWG on telomere length of cfDNA, which could represent a molecular mechanism underpinning the effects of maternal behaviours on foetal well-being.

## 1. Introduction

Gestational weight gain (GWG)—which depends on body composition, weight of the foetus, placenta and amniotic fluid [1]—represents a natural response to host the growing foetus. The US-based Institute of Medicine (IOM) has promoted the development of recommendations to identify an optimal amount of GWG according to the pre-pregnancy Body Mass Index (BMI) [1,2,3,4,5]. However, more than half of pregnant women do not respect clinical practice guidelines for weight gain [6,7,8]. The adherence to these guidelines is crucial to reduce the risk of adverse outcomes for mothers and their newborns [9,10,11,12,13]. With respect to IOM guidelines, both greater and lower weight gain contribute to short- and long-term health complications [2,14,15,16]. For instance, excessive GWG is associated with an increased risk of high blood pressure [17], diabetes [18], caesarean section [19], postpartum weight retention [20] and obesity [12]. As regard newborns born from mothers who gained weight excessively, they are more likely to be large for gestational age [18,21,22] and to develop metabolic disorders during adolescence and adulthood [23,24]. By contrast, low GWG is associated with increasing risks of pre-term delivery [25]. For all these reasons, there is the need for uncovering molecular mechanisms associated with GWG to identify mothers who could benefit more from preventive strategies.

In this scenario, telomere length represents a promising biomarker for biological aging and age-related diseases. Telomeres are repeating DNA sequences at the ends of chromosomes that progressively shorten with cell division [26]. To maintain genomic stability, telomeres protect the chromosomes from DNA damage and shorter telomeres are considered as a marker of the cumulative damage to which cells have been exposed [26]. Telomere integrity is maintained by the specific activity of telomerase, characterized by an enzymatic protein component that adds the telomeric DNA repeats at the end of chromosomes, and a telomerase RNA template for telomeric DNA synthesis [27]. In adults, shorter telomeres are associated with diabetes [28], cancer [29] and cardiovascular disease [30]. However, also before the onset of age-related diseases, obesity might contribute to cumulative burden of oxidative stress and chronic inflammation, accelerating the telomere shortening process. During pregnancy, adverse exposures such as maternal stress, smoking and higher levels of air pollution are associated with shorter telomeres measured in cord blood [31,32,33], placenta [34] and other children’s samples [35]. In particular, it has been demonstrated that maternal pre-pregnancy BMI was associated with telomere shortening in cord blood and placenta [36]. Some studies evaluated the association of telomere length in cord blood with preterm birth [37] and birth weight [38,39]. Moreover, since telomerase activity is time- and location-regulated in both embryo and placental tissues, associations between telomerase activity and pregnancy complications—such as intrauterine growth restriction—have been previously observed [40]. Circulating cell-free DNA (cfDNA) in plasma and serum has been proposed as a novel biomarker for prenatal diagnosis [41,42] and with applications in oncology [43,44]. Moreover, the relationship between cfDNA in serum and several diseases has raised much interest in investigating the role of cfDNA in other body fluids, such as urine [45], saliva [46] and amniotic fluid [47]. In line, amniotic fluid has been proposed as an alternative source of potential biomarkers for prenatal diagnosis [48]. Indeed, amniotic fluid surrounds the foetus with a continuous exchange with foetal organs and gestational tissues [49,50,51]. After removing the cellular components, the cell-free supernatant that remains reflects maternal and foetal well-being [51,52,53,54,55,56,57,58,59]. In particular, amniotic fluid contains a greater amount of cell-free foetal- and pregnancy-related DNA than maternal serum [60,61,62,63]. 

With these thoughts in mind, in order to study the relationship between telomere length and weight gain during pregnancy we used data and samples from the ongoing prospective “Mamma & Bambino” study, which enrolls mother–child pairs from Catania, Italy. Here, we report findings about the relationship between GWG and telomere length in maternal leucocyte DNA (mlDNA) and cfDNA of amniotic fluid.

## 2. Materials and Methods

### 2.1. Study Design

This study was conducted on samples and data obtained from mother-child pairs of the “Mamma & Bambino” cohort. It is an ongoing prospective study research with the aim of evaluating how the exposome affects mothers’ and children’s health [64,65,66,67]. Full details and protocols of the “Mamma & Bambino” cohort are described elsewhere [64,65,66,67]. In brief, the cohort consists of pregnant women attending for prenatal genetic counselling at the Azienda Ospedaliero Universitaria Policlinico “G. Rodolico -San Marco” (Catania, Italy). Those with multiple pregnancy, autoimmune and chronic diseases, pregnancy complications, intrauterine foetal death and congenital malformations are excluded. Women are generally recruited from 4 to 20 weeks of gestation and the study entails planned follow-ups at delivery and after two years birth. In the current analysis, we used data and samples from mothers who completed singleton pregnancy and with available data on GWG at delivery.

### 2.2. Assessment of Gestational Weight Gain

At recruitment, women were asked to report their height and pre-pregnancy weight to calculate pre-pregnancy body mass index (BMI) as kg/m^2^. Women were classified as underweight, normal weight, overweight or obese based on their pre-pregnancy BMI, according to WHO criteria [68]. Firstly, maternal weight achieved at recruitment was calculated by subtracting the self-reported pre-pregnancy weight from the weight at recruitment. Next, maternal weight achieved at delivery was collected from clinical records and total GWG was calculated by subtracting the self-reported pre-pregnancy weight from the weight at delivery. As described by the IOM guidelines [6], GWG was classified as reduced, adequate, or excessive according to pre-pregnancy BMI.

### 2.3. Covariate Ascertainment

Beyond anthropometric measures, our analysis considered several covariates that might affect GWG, telomere length and their relationship. At recruitment, demographics, socio-economic information and lifestyles were assessed by trained epidemiologists through structured questionnaires [64,65,66,67,69,70,71]. Maternal age and gestational ages at recruitment and at delivery were considered because of their potential effect on sampling and telomere length. In addition, we used the educational level and employment status as two proxy indicators of socio-economic status. Educational level was classified as low (having primary education), medium (having secondary education) or high (having tertiary education). Employment status was categorized as employment or unemployment, which also included students and housewives. We also categorized women in those who have previously had at least a child and those who have not. Regarding lifestyles, we assessed smoking status, daily energy intake and adherence to the Mediterranean Diet (MD). Specifically, dietary data were collected using a 95-item semiquantitative Food Frequency Questionnaire (FFQ). This tool was referred to 30 days before recruitment [72] and, for each item, it asked to report both frequency of consumption and portion size. Daily energy intake was calculated considering the table of food composition released by the US Department of Agriculture and adapted to typical Italian foods. Adherence to MD was evaluated using the Mediterranean Diet Score, as described in detail elsewhere [73].

### 2.4. DNA Extraction

Biological samples included maternal blood obtained at recruitment and an aliquot of amniotic fluid from women who underwent amniocentesis. Full details on protocols of DNA extraction are reported elsewhere [67,71]. Genomic mlDNA was extracted from 200 μL of maternal blood, while cfDNA was extracted from the supernatant of amniotic fluid obtained after centrifugation. DNA extraction and purification were performed using the QIAamp Blood Kit (Qiagen, Milan, Italy) on the QIAcube instrument (Qiagen, Milan, Italy), as described by the manufacturer’s protocol. Concentration and purity of DNA were assessed by NanoDrop 1000 spectrometer and by Qubit 3.0 Fluorometer using dsDNA HS Assay Kit (Thermo Fisher Scientific, Carlsbad, CA, USA).

### 2.5. Relative Telomere Length

Relative telomere length was measured by real-time quantitative polymerase chain reaction (qPCR), using the Relative Human Telomere Length Quantification Assay Kit (ScienCell Research Laboratories, Carlsbad, CA, USA). The qPCR was performed on a QuantStudio 7 Flex Real-Time PCR System (Thermo Fisher Scientific, Carlsbad, CA, USA), according to the manufacturer’s protocol. The following sets of primers were used: the telomere (T) primer set amplified telomere sequences; the single-copy reference (S) primer set amplified a 100 bp-long region on human chromosome 17 and was used as reference for data normalization. The specificity of these primer sets was validated by the manufacturer through qPCR with melt curve analysis. Each reaction contained 1 μL of DNA (5 ng/μL), 2 μL of primer solution (telomere or SCR), 10 μL of 2X GoldNStart TaqGreen qPCR master mix (ScienCell Research Laboratories, Carlsbad, CA, USA) and 7 μL of nuclease-free water. The PCR conditions were as follows: denaturation (95 °C for 10 min); 32 cycles of 95 °C for 20 s, 52 °C for 20 s and 72 °C for 45 s. The qPCR was calibrated including in each plate a serial dilution of DNA from randomly selected samples. All reactions were run in duplicate and relative telomere length was expressed as the average of telomere/single copy reference (T/S) ratio.

### 2.6. Statistical Analysis

Statistical analyses were performed using SPSS v.25. Descriptive statistics was initially performed using frequencies (percentage, %) or median and interquartile range (IQR) due to the skewness of quantitative variables. Bivariate analyses were conducted using the Mann–Whitney or the Kruskal–Wallis tests for quantitative variables and the Chi-squared test for trend for categorical variables. Relative telomere length was also plotted against weight gain at recruitment and at delivery to inspect linear or non-linear relationships. Next, we plotted relative telomere length by the tertile distribution of GWG, as well as by its classification in reduced, adequate, or excessive. We also applied a logistic regression model using adequate GWG as dependent variables and the following covariates: relative telomere length, maternal age, gestational age at recruitment, educational level, having children, pre-pregnancy BMI, total daily energy intake and gestational age at delivery. The adjusted association of relative telomere length with adequate GWG was reported as β coefficient and its Standard error (SE). All tests were two-sided and performed at a significance level α = 0.05.

## 3. Results

### 3.1. Characteristics of Study Population

The current analysis included 270 mothers who completed singleton pregnancy, with available information on GWG at delivery. Table 1 describes the characteristics of the study population and their comparison across categories of GWG (i.e., 101 women gained weight adequately, while 91 and 78 reported reduced and excessive GWG, respectively). As expected, there were strong relationships between maternal anthropometric measures and GWG. Indeed, women with adequate GWG were those with the lowest pre-pregnancy weight and BMI. By contrast, weight at delivery increased from reduced to excessive GWG categories. A similar trend was observed for education, so that the proportion of women with low or medium educational level increased from reduced to excessive GWG. Moreover, the proportion of women who already had at least one child before the current study was higher in those with adequate GWG than their counterparts. With respect to dietary habits, we did not find any association with adherence to Mediterranean Diet (MD), but total daily energy intake increased across GWG categories.

### 3.2. Relationship of Telomere Length with Maternal Characteristics

Among recruited women, we collected 252 maternal blood samples used to analyse telomere length of mlDNA. Notably, relative telomere length of mlDNA did not correlate with maternal age, pre-pregnancy BMI, total energy intake, Mediterranean Diet Score (MDS) and gestational age at sampling and at delivery (*p*-values > 0.05). Moreover, relative telomere length did not differ across categories of educational level, employment, smoking status, parity and pre-pregnancy BMI (*p*-values > 0.05). 

We also obtained 150 samples of amniotic fluid from those who underwent amniocentesis. These samples were used to evaluate telomere length of cfDNA. Relative telomere length of cfDNA and mlDNA did not correlate with each other (*p* > 0.05). On the contrary, relative telomere length of cfDNA was negatively but weakly correlated with gestational age at sampling (Spearman coefficient = −0.152; *p* = 0.046) and positively with total energy intake (Spearman coefficient = 0.157; *p* = 0.038). No correlations were evident with the remaining maternal characteristics, as well as with birth length and birth weight (*p*-values > 0.05). Relative telomere length did not also differ across categories of educational level, employment, smoking status, parity, pre-pregnancy BMI, type of delivery and newborn gender (*p*-values > 0.05).

### 3.3. Relationships between Gestational Weight Gain and Telomere Length

**Figure 1:**
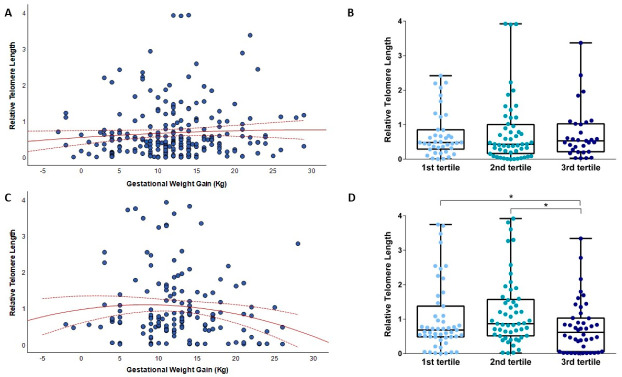
The relationship of gestational weight gain with relative telomere length. (**A**) shows the relationship of gestational weight gain with telomere length of mlDNA; (**B**) shows the box plots of telomere length of mlDNA by the tertile distribution of GWG; (**C**) shows the relationship of gestational weight gain with telomere length of cfDNA from amniotic fluid; (**D**) shows the box plots of telomere length of cfDNA by the tertile distribution of GWG. * *p*-value < 0.05 based on the Mann–Whitney or Kruskal–Wallis test.

We next evaluated the relationship of relative telomere length in mlDNA and cfDNA with gestational weight gain (Figure 1). To do that, we also classified women according to the tertile distribution of GWG: first tertile from −2 to 9 Kg; second tertile from 10 to 13 Kg; third tertile from 14 to 28 Kg. As depicted in Figure 1A, relative telomere length of mlDNA seemed to weakly increase with GWG. However, Figure 1B did not show a significant difference according to the tertile distribution of GWG (*p* = 0.559). By contrast, Figure 1C suggested a U-shaped relationship between GWG and relative telomere length of cfDNA. The U-shaped relationship was confirmed by the comparison of relative telomere length across tertiles of GWG (*p* = 0.016; Figure 1D). In particular, women in the third tertile showed shorter relative telomere length than those in the second tertile (*p* = 0.014; Figure 1D). 

**Figure 2:**
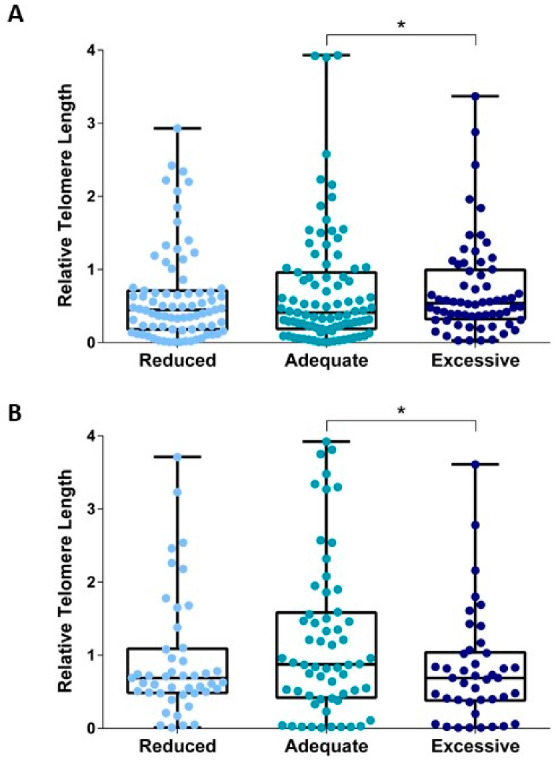
The relationship between categories of gestational weight gain and relative telomere length. (**A**) shows the box plots of telomere length of mlDNA according to GWG categories; (**B**) shows the box plots of telomere length of cfDNA according to GWG categories. * *p*-value < 0.05 based on the Mann–Whitney test.

**Figure 3:**
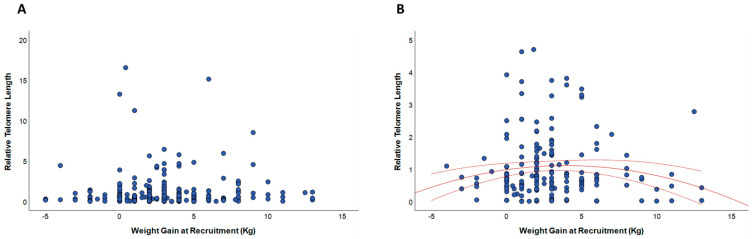
The relationship of weight gain at recruitment with relative telomere length. (**A**) shows the relationship of weight gain at recruitment with telomere length of mlDNA; (**B**) shows the relationship of weight gain at recruitment with telomere length of cfDNA from amniotic fluid.

We next compared relative telomere length across categories of GWG that considered reduced, adequate, or excessive weight gain during pregnancy. Regarding mlDNA, we showed longer telomere length in women with excessive GWG that in those who gained weight adequately (*p* = 0.017; Figure 2A). By contrast, telomere length of cfDNA was lower in amniotic fluid from women with reduced or excessive GWG, if compared with those who gained weight adequately (Figure 2B). Yet, the difference was statistically significant for women with excessive GWG (*p* = 0.044) but not for those with reduced GWG (*p* = 0.117; Figure 2B). It is also worth mentioning that the relationship was already evident if considering weight gain at recruitment (Figure 3).

**Figure 4:**
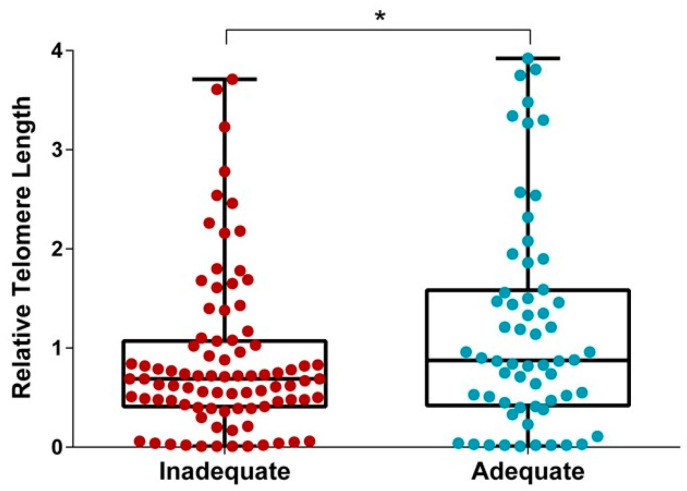
The comparison of telomere length of cfDNA from amniotic fluid between adequate and inadequate gestational weight gain. * *p*-value < 0.05 based on the Mann–Whitney U Test.

We next compared relative telomere length between women who gained weight adequately and those who did not. This comparison showed higher relative telomere length of cfDNA in women with adequate GWG (*p* = 0.017; Figure 4). Finally, we applied a logistic regression model including other maternal characteristics (i.e., age, gestational age at sampling, educational level, parity, pre-pregnancy BMI, total daily energy intake, gestational age at delivery) that might affect telomere length and/or GWG. Interestingly, the association between cfDNA telomere length and adequate GWG was significant, after adjusting for the abovementioned maternal characteristics (β = 0.464; SE = 0.189; *p* = 0.014).

## 4. Discussion

In the present study, we show for the first time a link between relative telomere length and GWG, though the shape of the relationship depends on DNA source. In particular, we observed a U-shaped relationship when analysing cfDNA in amniotic fluid, with longer relative telomere length in samples from mothers who gained weight adequately. To place our results in context, it is worth mentioning that a meta-analysis of nearly 120,000 subjects suggested an inverse association between obesity and telomere length [29]. This is in line with a collaborative cross-sectional meta-analysis of 87 observational studies and 146,114 individuals, showing a 3.99 bp decrease in telomere length for each unit increase in BMI [74]. However, these analyses indicated a high degree of heterogeneity across studies and an overall lack of evidence on pregnant women [29,74]. This heterogeneity could, at least in part, explained by the effect of chronological age on telomere length. In fact, it has been greatly demonstrated how telomere length decreased with increasing chronological age [75,76]. However, in our study, we did not find a correlation between maternal age and telomere length of mlDNA and cfDNA. Yet, during the gestational period, maternal body could experience changes at cellular and molecular levels that might prevent the relationship between age and telomere length [77]. For instance, it has been proposed that gestational age, rather than chronological age, could influence telomere shortening in placental DNA [78]. Moreover, other factors could interact with and/or mediate this relationship.

To our knowledge, only few studies investigated the effect of maternal pre-pregnancy BMI on telomere length, and none focused on maternal GWG. For instance, Martens and colleagues reported a decline in newborns’ telomere length with increasing maternal pre-pregnancy BMI, as assessed in both cord blood and placental tissues [36]. Interestingly, this effect seemed to persist in childhood, as demonstrated by Clemente and colleagues [79]. The authors used data from the Human Early-Life Exposome (HELIX) study to demonstrate that child’s leukocyte telomere length decreased with increasing maternal pre-pregnancy BMI [79]. However, the role of telomerase in the association between increased BMI and shortened telomere length is not well investigated. Epel and colleagues described reduced telomerase activity with increasing BMI in healthy women, which may be an important factor for the observed relationship between shorter telomeres and body weight. Further research is needed to evaluate whether the relationship between telomerase activity and BMI in pregnant women could be associated with altered neonatal telomerase activity [80].

We add to this knowledge, suggesting the influence of maternal weight gain during pregnancy on telomere length of cfDNA from amniotic fluid. The observed difference, though small, could add motivations to keep studying the effect of weight gain on aging biomarkers. Of note, the relationship remained significant after controlling for the potential effects of covariates (i.e., age, gestational ages at recruitment and at delivery, educational level, previous pregnancies, pre-pregnancy BMI and total daily energy intake). Interestingly, the effect of maternal weight gain on telomere length of cfDNA was already evident in early pregnancy (i.e., considering GWG at a median gestational age of 16 weeks). This was consistent with the Developmental Origins of Health and Disease (DOHaD) hypothesis, for which prenatal environment programs the foetus for challenges that it is likely to experience after birth [81]. For instance, it has been proposed a potential relationship between epigenetic mechanisms (i.e., DNA methylation) and telomere attrition rate in early life, which in turn could be influenced by internal and external stressors [82]. However, it is necessary to investigate if even small differences in telomere length could be associated with pregnancy and neonatal outcomes. In this scenario, identifying activators of telomerase that could complement the benefits of a healthy lifestyle will be an important field of research in the ongoing evaluation of the telomere system [83].

In line, there is the current need for developing non-invasive tests to understand foetal well-being. These tests should be based on maternal serum or urine, avoiding invasive tests such as amniocentesis. Yet, more than 80% of cfDNA fragments in the maternal serum are short and fragmented [84]. Compared to maternal serum, amniotic fluid contains a much greater concentration of cfDNA [85], which is largely uncontaminated by maternal- and trophoblast-derived nucleic acids. Thus, amniotic fluid represents a relatively pure foetal sample and its supernatant is a valuable and widely available but under-utilized resource [86]. It was not our intention to prefer amniotic fluid over maternal blood for the analysis of relative telomere length. However, in future, it will be interesting to evaluate if foetal DNA from maternal blood reflects the same difference observed in our study. 

Our findings also provide further motivation to study telomere length and telomerase activity as potential molecular mechanisms underpinning the effects of maternal behaviours on the development of chronic disease later in life. Although mechanisms by which inadequate weight gain affects telomere length are not yet fully understood, it is plausible that they rely on a chronic inflammatory and oxidative state in utero [87]. Despite this speculation, however, we currently need more experimental work to better understand how maternal weight gain affects telomere dynamics in the foetus. 

Our study had some limitations that should be considered. Firstly, the information on pre-pregnancy weight was self-reported, which cannot completely exclude a potential reporting bias. It is also true, however, that previous studies demonstrated how self-reported pre-pregnancy weight correlated with that measured [88,89]. Secondly, we worked on total GWG reached at delivery without accounting for weight trajectories throughout pregnancy. Yet, our analysis at the time of recruitment already showed an effect of maternal weight gain on telomere length of cfDNA. Although we observed an early influence of GWG on telomere length, our analysis did not focus on their causal–effect relationship. Thirdly, although amniotic fluid seems a relatively pure foetal sample, a low proportion of cfDNA from placenta cannot be completely excluded [90]. Moreover, we assessed relative telomere length by qPCR, which has higher assay variability than terminal restriction fragment analysis [91]. Finally, the presence of residual confounders cannot be completely ruled out, such as that deriving from fatherly influence [92,93]. 

## 5. Conclusions

In conclusion, we found that relative telomere length of cfDNA is associated with maternal weight gain during pregnancy and at delivery. This suggests an early influence of GWG on telomere length, which could represent a molecular mechanism underpinning the effects of maternal behaviours on foetal well-being. However, further experimental studies are needed to biological events that regulate this relationship and to consider other factors influencing the uterine environment during pregnancy.

## Figures and Tables

**Table 1:** Characteristics of women from the “Mamma & Bambino” cohort (*n* = 270) according to gestational weight gain categories.

Characteristics	Overall (*n* = 270)	Reduced GWG (*n* = 91)	Adequate GWG (*n* = 101)	Excessive GWG (*n* = 78)	*p*-Value ^a^
Age ^b^	37.0 (4.0)	37.0 (4.0)	38.0 (4.0)	37.0 (4.0)	0.699
Gestational age at sampling ^b^	16.0 (4.0)	16.0 (4.0)	16.0 (3.0)	16.0 (2.0)	0.953
Educational level (%)					
Low	17.8%	16.5%	16.8%	20.5%	0.038
Medium	47.8%	40.7%	45.5%	59.0%	
High	34.4%	42.8%	37.7%	20.5%	
Working (%)					
Employment	57.4%	54.9%	61.4%	55.1%	0.593
Unemployment	42.6%	45.1%	38.6%	44.9%	
Smokers (%)	20.5%	15.4%	20.0%	27.3%	0.216
Having children (% yes)	67.7%	64.3%	76.8%	59.7%	0.041
Total energy intake ^b^	1750 (620)	1667 (674)	1752 (545)	1858 (596)	0.045
MDS ^b^	4.0 (2.0)	4.0 (2.0)	4.0 (2.0)	4.0 (2.0)	0.102
Pre-pregnancy weight ^b^	61.0 (15.2)	62.0 (16.0)	59.0 (13.0)	64.5 (18.3)	0.012
Pre-pregnancy BMI ^b^	22.8 (5.1)	22.8 (4.8)	22.0 (3.8)	25.0 (5.7)	0.002
Pre-pregnancy BMI categories					
Underweight	6.7%	6.6%	6.9%	6.4%	<0.001
Normal weight	64.1%	68.1%	77.2%	42.3%	
Overweight	17.4%	9.9%	8.9%	37.2%	
Obese	11.9%	15.4%	7.0%	14.1%	
Weight at delivery ^b^	74.0 (15.0)	68.5 (11.5)	73.0 (12.7)	82.0 (15.2)	<0.001
Gestational age at delivery ^b^	39.0 (2.0)	38.0 (2)	39.0 (2)	39.0 (2.0)	0.383

^a^*p*-values are based on the Kruskal–Wallis test for quantitative variables, or chi-squared test for categorical variables ^b^ Data are reported as median and interquartile range (IQR). Abbreviations: GWG, gestational weight gain; MDS, Mediterranean Diet Score; BMI, body mass index.

## Data Availability

The datasets analysed during the current study are available from the corresponding author on reasonable request.
